# A Pilot Study of Stress System Activation in Children Enrolled in a Targeted Prevention Program: Implications for Personalization

**DOI:** 10.3390/ijms19020361

**Published:** 2018-01-25

**Authors:** Bonnie Klimes-Dougan, David A. Klingbeil, Alaa Houri, Kathryn R. Cullen, Meredith Gunlicks-Stoessel, Gerald August

**Affiliations:** 1 Department of Psychology, University of Minnesota, N412 Elliot Hall, 75 E. River Road, Minneapolis, MN 55455, USA; 2 Department of Educational Psychology, University of Wisconsin-Milwaukee, 2400 Hartford Ave., Milwaukee, WI 53211, USA; davidak5@uwm.edu; 3 Department of Educational Psychology, University of Minnesota, 250 Educational Sciences Bldg, 56 East River Road, Minneapolis, MN 55455, USA; houri005@umn.edu; 4 Department of Psychiatry, University of Minnesota, F282/2A West Building 2450, Riverside Avenue South, Minneapolis, MN 55454, USA; rega0026@umn.edu (K.R.C.); mgunlick@umn.edu (M.G.-S.); 5 Department of Family Social Sciences, University of Minnesota, 290 McNeal Hall, 1985 Buford Avenue, St. Paul, MN 55108, USA; augus001@umn.edu

**Keywords:** children, prevention, intervention, cortisol, HPA axis, personalization

## Abstract

Empirically validated interventions addressing childhood psychological problems are now readily available, but success likely depends in part on accurately identifying which children will benefit from which intervention. This pilot study examined the stress activation and response system, first as a way to differentiate high versus low-risk children, and second to explore indicators of the stress system associated with favorable intervention response. Method. Participants (*N* = 43, 58% male) were school-aged children who qualified for inclusion in the Early Risers “Skills for Success” Prevention Program based on their elevated levels of aggressive and/or socially withdrawn behavior and a normally developing comparison group. Compared to the normally developing group, children who were participants in the intervention exhibited a more blunted cortisol response to the stress paradigm. However, for the children in the intervention group, elevated cortisol levels at the start of the stress paradigm were concurrently associated with internalizing problems and predictive of improvement in internalizing problems over time. These findings provide preliminary evidence that hypothalamic pituitary adrenal (HPA) axis biological variables may be helpful tools for identifying children who would benefit from intervention and personalizing interventions.

## 1. Introduction

Early intervention delivered in community systems of care (e.g., schools) is critical to prevent severe psychiatric problems [1] particularly interventions that take place in childhood or adolescence. Not only does psychopathology often begin during this developmental period but efforts to intervene early may be more successful due in part to the enhanced plasticity of critical biological systems. A number of evidence-based preventive interventions (EBIs) have been developed and validated for use with children and adolescents suffering from psychological problems, yet typically about a third to a half of those participating in EBIs fail to show a favorable response [2]. The cost is considerable to individuals and larger society for unsuccessful interventions [3]. However, currently, clinicians have little empirical evidence to guide their decisions about which treatment would be most efficacious for a particular individual. Part of the variability of intervention response may be due to individual differences in disorder presentation. Therefore, continued work is needed to determine who might be more or less likely to benefit from a particular treatment by identifying predictors and moderators of EBI response [4]. Ultimately, this line of research will facilitate new directions for therapeutic innovation, including the emergence of personalized interventions [5].

Identifying biological indices that predict intervention response holds considerable promise [6]. During childhood, features of the stress system become increasingly consolidated [7], yet remain responsive to the ameliorative impact of interventions [8]. The hypothalamic pituitary adrenal (HPA) axis represents a critical aspect of the biological stress system functioning through the release and regulation of hormones, including cortisol. Healthy adaptation and survival rely on an individual’s ability to produce increased levels of cortisol when confronted with danger. This elevation in cortisol serves to mobilize the physical and cognitive resources that enable the individual to effectively combat the stressor [9]. However, both heightened and blunted reactivity of the HPA axis in response to a stressor can be problematic. Hyper-arousal of the HPA axis is commonly associated with internalizing psychopathology (i.e., anxiety, depression), while hypo-arousal of the HPA axis is associated with externalizing psychopathology (i.e., Attention Deficit Disorder; Oppositional Defiant Disorder) [10]. Children that require early intervention often represent a heterogeneous group (e.g., with some primarily exhibiting internalizing problems and others showing high co-morbidities with externalizing problems) [11]. Importantly, researchers have found greater HPA axis activation to be correlated with better response to interventions in both children with internalizing and externalizing psychopathology [12]. For example, externalizing boys with elevated cortisol levels were significantly more responsive to therapeutic interventions [13]. Similarly, for adolescent girls with depressive problems, elevated HPA axis activation within the context of a conflict discussion task predicted more favorable Interpersonal Psychotherapy treatment outcomes [14].

The purpose of this pilot study was to use a multiple levels of analysis approach [15] to assess stress system functioning in children with elevated socially withdrawn and/or aggressive behavior who were enrolled in Early Risers Skills for Success Prevention Program, an evidence-based, targeted prevention program [16]. The first aim was to evaluate stress system functioning, including physiological functioning, self-reported ratings of experienced stress, and observer ratings of expressed stress in the context of the Trier Social Stress Test—Child Version (TSST-C) in young, school-aged children. Overall, there is a paucity of work considering stress system functioning in this age group. However, there is some evidence showing divergent patterns HPA axis functioning for those with typical and atypical development [17]. Given the adaptive function of stress responding, we predicted elevations across all three indexes would be evident in the typically developing young children who were considered normal controls (NC). Second, we evaluated if stress system functioning was concurrently and predictively related to internalizing and externalizing problems for high-risk children enrolled in the Early Risers Skills for Success Prevention Program (ER). We predicted that elevated levels of stress system functioning may be concurrently and predictively related to better adaptation.

## 2. Results

Table 1 summarizes the demographic information and shows results of self-report and teachers’ ratings of psychological problems. As shown in Table 2, the results suggest that the TSST-C was successful at eliciting a stress response as indicated by a within-subject effect for changes in cortisol levels, *F*(3.18, 127.50) = 2.56, *p* = 0.05; within-subject effects for experienced stress *F*(2.96, 147.37) = 18.19, *p* < 0.001, and moderate rates of expressed (observed by experimenter) stress during the speech task (46.5%), the arithmetic task (49%), and minimal stress upon having completed the tasks (92% of the participants appeared pleased to have the task complete).

To address the first aim of this study, we considered if there were group differences between ER and NC. The results for area under the curve initial (AUCi) (but not area under the curve ground [AUCg], self-reports, or observed ratings) revealed a main effect for group, *F*(1, 37) = 4.20, *p* = 0.04. The NC group exhibited a larger relative elevation of cortisol from their pretest levels than the ER group (Table 2).

The second study aim addressed the concurrent and predictive links between cortisol and psychological problems. The results showed some evidence of concurrent associations between stress response and psychological problems. AUCg (but not AUCi, self-reports, or observed ratings) was correlated with teacher reported internalizing problems (*r* = 0.39, *p* = 0.04) for the ER group at Year 1 (Y1), primarily due to correlations between CORT1 (*r* = 0.40, *p* = 0.04) and CORT2 (*r* = 0.44, *p* = 0.02) and internalizing problems. There were no significant correlations for cortisol measures with externalizing problems at Y1.

Finally, relevant to identifying possible predictors of intervention, we considered if HPA axis functioning was associated with improvement in functioning in participants involved in ER training from Y1 to year 2 (Y2). We found that AUCi (but not AUCg, self-reports or observed ratings) was associated with the BASC-2-TRS internalizing change score (*r* = −0.45, *p* = 0.05) for the ER group. In other words, a greater drop in cortisol over the course of the TSST-C was associated with a positive prognosis (i.e., improvement in internalizing symptoms from the Y1 to Y2 based on the teacher’s report). Follow-up analysis indicates that the difference between CORT5 and CORT1 largely accounted for this finding (*r* = −0.55, *p* = 0.01), suggesting that those with slightly elevated CORT1 values with a linear decline across the TSST-C assessment had the most favorable response to ER.

## 3. Discussion

There is growing enthusiasm for adopting an explicit developmental psychopathology approach to prevention by incorporating biological markers for assessing and monitoring intervention efficacy, as well as identifying indices that moderate intervention response to facilitate efficient matching of individuals to interventions [6]. The results highlight the feasibility and potential advantages of using a multi-level approach to evaluate a stress for children at risk for mental health problems who are enrolled in an intervention trial. The findings should be considered preliminary. Nevertheless, the results suggest that biological factors differed between high- and low-risk groups and were concurrently associated with internalizing symptoms. Most importantly, results indicate that byproducts of the HPA axis (a pattern characterized by hyperarousal early on in the TSST-C procedure followed by a precipitous decline) may be a predictor of a favorable treatment response. These results are broadly consistent with other work with youth that has shown that hyperarousal predicted more favorable intervention responses [14] and other evidence suggests that intervention may have prevented the attenuation of cortisol levels associated with externalizing problems [12]. It is possible that hyperarousal is a common predictor of a range of clinical interventions given that recently our group noted some evidence of elevations in TSST in depressed adolescents who show more favorable responses to psychopharmacological treatment [18]. However, a deeper understanding of stress system functioning across development is needed as this pattern does not typically hold for depressed adults undergoing psychosocial or psychopharmacological treatment [19]. There are some aspects of stress system functioning that do not seem to be associated with intervention outcomes including indices of self-reported experienced stress, experimenter-rated expressed stress, and basal cortisol for these participants that was collected at the end of the school day [20]. More evidence is needed in a related line of research that evaluates if changes to stress system functioning correspond to intervention outcomes [21].

There are a number of important steps still needed to identify factors that may guide in treatment selection for individual children suffering from psychological problems. Most importantly, large-scale, randomized control trials with long-term follow-up assessments are needed to fully test for moderators of treatment response that are relevant to personalization. Biological factors that predict intervention response may prove to be common factors for a range of different treatments. Thus, it is possible that these findings in isolation may have limited value ultimately to personalization. An important next step would be to conduct trials that consider two or more EBIs, for, ultimately, it will be important to identify both common predictors as well as unique predictors (e.g., predict psychosocial but not psychopharmacological) of intervention response. Additionally, it would have also been preferable to follow the NC group for the subsequent assessments to determine the relative change in internalizing and externalizing symptoms over time as they relate to physiological changes. Recommendations for future work also include (a) more comprehensive assessments of the physiological stress system (e.g., broader indexes of the HPA cascade, limbic system activation); (b) a broader index of child problems; (c) the systematic assessment of a broader range of possible confounds (e.g., circadian cycles, medication use, caffeine use, exposure to tobacco in the home, etc., should have been more systematically assessed and considered as covariates), and alternative assessment windows (e.g., this study began after the first year of ER programming at a point when significant improvement in symptoms had already started to take place). Finally, the study questions would be ideally assessed using more comprehensive analytic models (e.g., Structural Equation Modelling) that account for a range of variables within the same model. Unfortunately, the small sample precluded the applicability of these more integrative analytic approaches.

## 4. Method

### 4.1. Participants

This study was approved by the Institutional Review Board at the University of Minnesota. Parents provided consent and children provided assent prior to study participation. Children from both groups participated in after school programming. Child participants were compensated with a small toy (worth approximately $10) of their choice. Participant who qualified for inclusion in the Early Risers “Skills for Success” Prevention Program (ER) were selected for the program based on the presence early problem development (*n* = 27) based on subclinical to clinical elevations on the aggressive and socially withdrawn behavior scales of the Behavioral Assessment System for Children-2-Teacher Report Scale (BASC-2-TRS). The normal control group (NC) was comprised of typically developing children who participated in other available after school programming, which included supervised snack, homework period, arts and crafts, and playground time (*n* = 16). This study focuses on assessments at Year 1 (Y1) and Year 2 (Y2) for ER but only Y1 for NC. This pilot study was conducted in three schools in one region of Minnesota, and was part of a larger ER intervention trial, implemented with fidelity across 27 sites/regions in rural Minnesota from 2005 to 2009, which included baseline, Y1 and Y2 [22,23].

### 4.2. Measures

The BASC-2 is a commonly used, multidimensional system, using a four-point scale (ranging from 0 = never to 3 = almost always) for 139 items, to assess broad domains of externalizing, internalizing and school problems (αs = 0.85–0.94). Gender-specific normative scores are provided in the form of T-scores with a mean of 50 and a standard deviation of 10. Additional evidence for reliability and validity were reported by [24]. Child participants in both the ER and NC completed the Behavioral Assessment System for Children Self Report of Personality (BASC-2-SRP) at Y1. In this study, we focused on the Emotional Symptom Index (α = 0.94), which is composed of four scales indicating internalizing problems and two scales indicating the lack of personal adjustment (e.g., self-esteem and self-reliance). Additionally, ER participants’ primary classroom teachers completed the Teacher Rating Scales (BASC-2-TRS); Internalizing and Externalizing symptoms at the Y1 and Y2, as well as change scores for the Internalizing and Externalizing scales from Y1 to Y2 [24]. At Y1, ER and NC participants completed a slightly modified version of the Trier Social Stress Test—Child Version (TSST-C) that involved a 3-min preparation period, and a 5-min public speaking task (a story telling task) and a 5-min mental arithmetic task in front of a microphone, video recorder and two experimenters [17]. Five samples of saliva were obtained from each child, beginning with the pretest assessment prior to the start of the TSST-C (CORT1), immediately following the TSST-C (CORT2), and approximately every 15 min after the completion of the TSST-C (CORT3, CORT4, CORT5). Standard methods were used for saliva extraction and storage. Analysis was conducted by the Universität Trier in Trier, Germany (intra- and inter-assay variability coefficients of between 4.0–6.7% and 7.1–9.0%) [25] Primary analyses for physiological indices of stress included the use of individual values (e.g., CORT1) but primarily relied on commonly used summary scores of (a) area under the curve ground (AUCg), which is an index of overall cortisol levels and (b) area under the curve initial (AUCi), which is an index accounting for the rise in cortisol levels [26].

Child self-reports of experienced stress and experimenter ratings of expressed stress were obtained within the context of the TSST-C (similar to that reported by [27]. Immediately after the TSST-C, child participants were asked to answer a series of questions (read aloud by the experimenter with accompanying illustrations) to indicate degree of their level of stress from 1 (fine/calm) to 4 (high stress) for the period when they were (a) preparing to give the speech; (b) giving the speech; and (c) completing the subtraction task. During the TSST-C, the two experimenters completed ratings on a 1 (not at all stressed) to 6 (discontinued the procedure because appeared excessively stressed) scale for: (a) how stressed did the child appear stressed during the story telling task (ICCs = 0.814); and (b) how stressed did the child appear during the arithmetic task (ICCs = 0.720). The lead experimenter also reported yes or no to whether the child seem pleased to have successfully completed the task.

### 4.3. Prevention Programming

Children who showed elevated rates of externalizing and/or internalizing problems participated in two to three weekly sessions of Early Risers “Skills for Success” Prevention Program, a well-validated early intervention program [16] that was implemented with fidelity [23]. It is designed to (a) enhance children’s emotional and behavioral self-regulation, social adaptation, and academic success and (b) promote parents’ capacity to support their child’s healthy development by building positive parent-child relations, improving parenting practices, and increasing parent involvement in the child’s education. Briefly, the program is a theory-based, early-age-targeted preventive intervention for children at risk for the development of serious conduct problems or drug abuse. The ER program includes four individual components that are delivered over a two-year period. A key aspect of this program targets enhancing students’ stress regulation and problem solving skills using Promoting Alternative Thinking Strategies (PATHS) program [28], as well as literature appreciation, creative activities, and school support. Parents are invited to participate in education sessions and individualized family support services to further promote children’s adaptive skills. All three schools at this site/region shared a program implementer who led or co-led the after school programming.

### 4.4. Analytic Plan

A series of general linear model (GLM) analyses were conducted to examine possible differences between ER and NC group participants in physiological reactions, expressed stress, and experienced stress. Additionally, we conducted Pearson correlations to assess concurrent and predictive links of physiological stress reactions and symptomatology or changes in symptomatology over time. When analyses showed significant links with summary variables (e.g., AUCg), follow up analyses were conducted on specific assessment points (e.g., CORT1). All analyses were conducted in SPSS (Version 21, Chicago, IL, USA).

## 5. Conclusions

In conclusion, these results add to the existing studies on typically developing young school-aged children showing a pattern of rallying physiological resources in response to stressors. The results also begin to address some of the important questions relevant to personalization. This pilot study adds to the existing evidence for the utility of biological indices of stress reactivity and recovery for determining problem status, prognosis, and treatment planning. That is, these results show that it may be possible to identify biological predictors that are associated with a favorable response to long-term intervention. This line of research can be integrated into care models when empirically-derived decision rules (algorithms) can be applied to match clients to the type and/or dosage of intervention that optimizes their response [29]. A new generation of translational research is paving the way to enhance the likelihood that intervention can be as effective as possible by identifying the biological underpinnings of psychopathology. There is growing hopefulness that this line of work could provide information on developments of personalized interventions.

## Figures and Tables

**Table 1:** Demographic and psychopathology data for participants by group status.

Measure	Overall		Early Risers		Normal Control				
**Year 1**	***M*/*n* (%)**	**SD**	***M*/*n* (%)**	**SD**	***M*/*n* (%)**	**SD**	***t*/*χ*^2^**	***df***	***p***
Age	8.39	1.00	8.36	0.95	8.43	1.11	0.208	27.86	0.618
Sex (Male)	*n* = 25 (58%)		*n* = 18 (67%)		*n* = 7 (44%)		2.17	1	0.141
Race (Caucasian)	*n* = 42 (98%)		*n* = 26 (96%)		*n* = 16 (100%)				
Time of wake up	7:11	0:31m	7:11	0:38m	7:11	0:23m	0.04	26	0.966
Time of the stressor (CORT1)	15:51	0:38m	15:52	0:43m	15:50	0:30m	0.108	41	0.906
Race of experimenter (Caucasian)	*n* = 32 (74%)		*n* = 24 (89%)		*n* = 8 (50%)		0.359	1	0.054
BASC-2-SRP Emotional Symptoms Index	49.74	12.00	53.67	12.85	43.12	6.52	3.56	40.31	0.001
BASC-2-TRS Internalizing			58.42	12.36					
BASC-2-TRS Externalizing			60.00	10.24					
**Year 2**	***M*/*n* (%)**	**SD**	***M*/*n* (%)**	**SD**	***M*/*n* (%)**	**SD**	***t*/*χ*^2^**	***df***	***p***
BASC-2-TRS Internalizing			62.26	16.49					
BASC-2-TRS Externalizing			62.19	11.38					

BASC-2-SRP = Behavioral Assessment System for Children—Version 2—Self-Report of Personality; BASC-2-TRS = Behavioral Assessment System for Children—Version 2—Teacher Report Scale; *M*/*n* = Mean/number; SD = standard deviation; (*t*/*χ*^2^) = *t*-test/chi square test; *df* = degrees of freedom; *p* = probability; *n* = number; m = minutes.

**Table 2:** Salivary cortisol, ratings of experienced and expressed stress prior to, during, and following the Trier Social Stress Test—Child Version at Year 1.

Measure	Overall		Early Risers		Normal Controls					
	*M* (%)	SD	*M* (%)	SD	*M* (%)	SD	*F*	*df*	*p*	Partial *η*^2^
**Salivary Cortisol Values (µg/dL)**										
AUCi	0.30	7.94	−1.52	5.47	3.84	10.66	4.21	(1, 37)	0.047	0.102
AUCg	16.14	13.72	14.54	13.27	19.22	14.56	0.155	(1, 37)	0.696	0.004
CORT1	0.251	0.252	0.248	0.250	0.255	0.263	0.169	(1, 39)	0.684	0.004
CORT2	0.278	0.255	0.240	0.230	0.343	0.289	0.375	(1, 39)	0.544	0.010
CORT3	0.272	0.241	0.230	0.226	0.342	0.255	0.839	(1, 39)	0.365	0.021
CORT4	0.221	0.210	0.187	0.199	0.283	0.220	0.452	(1, 39)	0.505	0.012
CORT5	0.212	0.208	0.193	0.206	0.247	0.216	0.061	(1, 39)	0.807	0.002
**Experienced Stress**										
TSST-C Preparation	2.30	1.06	2.37	1.18	2.19	0.83	0.481	(1, 39)	0.492	0.012
TSST-C Speech	2.56	1.24	2.56	1.28	2.56	1.21	0.03	(1, 39)	0.864	0.001
TSST-C Math	1.91	0.90	1.81	0.92	2.06	0.85	0.054	(1, 39)	0.817	0.001
TSST-C Completion	1.07	0.34	1.11	0.42	1.00	0.00	0.503	(1, 39)	0.482	0.013
**Expressed Stress**										
TSST-C Speech	2.85	0.81	2.80	0.68	2.94	1.01	0.671	(1, 39)	0.418	0.017
TSST-C Math	2.83	0.85	2.72	0.68	3.00	1.08	0.061	(1, 39)	0.806	0.002
TSST-C Completion (yes, relieved)	90.7%		85.2%		100%					

µg/dL = micrograms per deciliter; AUCg = area under the curve ground; AUCi = area under the curve initial; CORT1 through CORT5 = first through the fifth salivary cortisol assay results; TSST-C = Trier Social Stress Test—Child Version; CORT; *M* = Mean; SD = standard deviation; *F* = F-test; *df* = degrees of freedom; *p* = probability; *η*^2^ = eta squared.
